# Editorial: Bridging the gap to molecular imaging and theranostics

**DOI:** 10.3389/fnume.2024.1385097

**Published:** 2024-04-11

**Authors:** Stuart More, Mike Sathekge, Vikas Prasad

**Affiliations:** ^1^ Division of Nuclear Medicine, Department of Radiation Medicine, University of Cape Town, Cape Town, South Africa; ^2^ Department of Nuclear Medicine, School of Medicine, University of Pretoria, Pretoria, South Africa; ^3^ Department of Nuclear Medicine, University of Pretoria and Steve Biko Academic Hospital, Pretoria, South Africa; ^4^ Division of Nuclear Medicine, Mallinckrodt Institute of Radiology, School of Medicine, Washington University in St. Louis, St. Louis, MO, United States

**Keywords:** FAPI imaging, biodosimetry and nuclear medicine, PET/CT, neuroendocrine tumors, breast cancer theranostics, hepatocellular carcinoma, nuclear cardiology, radiobiology

**Editorial on the Research Topic** Bridging the gap to molecular imaging and theranostics

With the current momentum and progress that nuclear medicine, molecular imaging, and radionuclide therapy has made over the last decade, it is not surprising that the field has grown considerably with ever new technologies, probes, and targets being discovered and investigated. The current research topic has gathered articles that have reviewed the intersection of the novelty of the field as well as reviewing some of the gaps that need to be addressed and investigated. With this in mind, the various authors in this topic have alluded to the infrequent but noteworthy applications of the field that require further exploration and translation into clinic care where applicable.

Fibroblast Activation Protein inhibitor (FAPI) has become a pharmaceutical of interest with respect to imaging the tumor microenvironment in a various number of oncological conditions (1). Its utility in non-oncological applications has also been explored in infectious, inflammatory, and rheumatological diseases. Nuclear cardiology applications have also been explored since the inception of this radiopharmaceutical. Gallium-68 FAPI ([^68^Ga] Ga-FAPI) was utilized in a study to predict myocardial dysfunction four months after patients had an initial myocardial event- the image of this article was recognized as SNMMI Image of the Year 2022 (2). It is against this background that Mpanya et al. explore the utilization of this novel tracer in a state-of-the-art review of using FAPI within nuclear cardiology to characterize various cardiovascular diseases other than ischemic heart disease, including hypertrophic obstructive cardiomyopathy (HOCM), amyloidosis, and sequalae of myocardial fibrosis.

Hepatocellular carcinoma (HCC) has shown an increase in incidence globally and, as such, new diagnostic and therapeutic targets need to be explored to address this disease entity. It is well described that 2-Deoxy-2-[^18^F]fluoro-d-glucose [2-[^18^F]FDG] has limitations with being able to characterize HCC owing to its variable uptake (3). Nyakale et al. in their review article on HCC, have evaluated the diagnostic performance of various imaging techniques as well as various tracers currently used to characterize this disease entity. As FDG PET is not as sensitive at detecting disease in HCC, Nyakale et al. specifically look at Prostate-Specific membrane Antigen (PSMA) and FAPI-labelled PET derivatives to characterize HCC in patients who may have metastatic disease and in preparation for potential radioligand therapy. A case is made for the utility of theragnostic applications in HCC, which include radioembolization(utilizing transarterial radionuclide agents such as Yttrium-90 microspheres and Rhenium-188 lipiodol) and cell-specific targets using PSMA and FAPI.

Theranostic applications within Pediatric Nuclear Medicine are not as well explored as the adult domain. Neuroendocrine tumors, albeit rare, do present in the pediatric population. Limited work has been presented in in pediatric patients, particularly utilizing peptide receptor radioligand therapy (PRRT) as a treatment option. Knowing that imaging and therapy in NETs are standard of care at present, Hlongwa et al. report on their experience in two pediatric patients who presented with metastatic NETs and therapy thereof, demonstrating the safety and efficacy of this treatment with the need to explore standardized guidelines and clinical trials for this population.

As theranostic applications in prostate cancer and neuroendocrine tumors are well established in the clinical care of patients with these conditions, new avenues should explore other malignancies that affect a significant portion of the global population, such as breast cancer. Vorster et al. review the current trends in characterizing the tumor microenvironment of breast cancer, including exciting targets such as chemokine receptor 4, bombesin antagonist, and integrin based imaging, exploring the challenges of the comprehensive implementation of diagnostic as well as theranostic approaches in this heterogenous disease.

Finally, the burgeoning role of radiobiology within the theranostic realm is ever growing and providing key understanding into the mechanism as to how tumor cells are targeted and the effectiveness of the various radiopharmaceuticals used in targeting these tumors. The need to be able to rationalize the safety and efficacy of various radiopharmaceuticals, assist in predicting response, and develop key biomarkers to enable follow up of patients needs to be actively explored (4). In their review article, Bolcaen et al. have explored the utility of biodosimetry and its potential routine implementation in clinical practice of nuclear medicine department. This is by utilizing various radiobiological assays in conjunction with image-based dosimetry to assist in more precise radioligand dose estimations. This will aid even further to maximize personalized medicine in the context of theranostics.

In conclusion, this Research Topic presents an array of articles aimed to highlight the current applications of nuclear medicine, molecular imaging, and theranostics in bridging the gap to future developments in this field.
